# 1270 nm near-infrared light as a novel vaccine adjuvant acts on mitochondrial photoreception in intradermal vaccines

**DOI:** 10.3389/fimmu.2022.1028733

**Published:** 2022-11-10

**Authors:** Yohei Maki, Toshihiro Kushibiki, Tomoya Sano, Takunori Ogawa, Eri Komai, Shusaku Takahashi, Etsuko Kitagami, Yusuke Serizawa, Ryosuke Nagaoka, Shinya Yokomizo, Takeshi Ono, Miya Ishihara, Yasushi Miyahira, Satoshi Kashiwagi, Akihiko Kawana, Yoshifumi Kimizuka

**Affiliations:** ^1^ Division of Infectious Diseases and Respiratory Medicine, Department of Internal Medicine, National Defense Medical College, Tokorozawa, Japan; ^2^ Department of Medical Engineering, National Defense Medical College, Tokorozawa, Japan; ^3^ Gordon Center for Medical Imaging, Department of Radiology, Massachusetts General Hospital, Charlestown, MA, United States; ^4^ Department of Global Infectious Diseases and Tropical Medicine, National Defense Medical College, Tokorozawa, Japan

**Keywords:** adjuvant, laser, light, mitochondria, vaccine, near-infrared, ROS - reactive oxygen species, ATP - adenosine triphosphate

## Abstract

With the development of laser technology in the 1960s, a technique was developed to inject intradermal vaccines immediately after irradiating the skin with laser light to elicit an adjuvant effect, referred to as “laser adjuvant.” We have been investigating the mechanism of laser adjuvant in influenza mouse models using noninvasive continuous-wave (CW) near-infrared (NIR) light mainly at a wavelength of 1064 nm, and have shown that the production of reactive-oxygen-species (ROS) in the skin and mast cells in the skin tissue plays an important role in the laser adjuvant effect. The new wavelength of 1270 nm NIR light is characterized by its ability to elicit the same vaccine adjuvant effect as other wavelengths at a lower energy, and may be suitable for clinical applications. In this study, we investigated the physiological activity of CW1270 nm NIR light in mast cells, its biological activity on mouse skin, and the durability of the vaccine adjuvant effect in influenza vaccine mouse models. We show that irradiation of mast cells with 1270 nm NIR light produced ROS and ATP, and irradiation of isolated mitochondria also produced ATP. In mouse skin, the relative expression levels of chemokine mRNAs, such as *Ccl2* and *Ccl20*, were increased by irradiation with 1270 and 1064 nm NIR light at minimum safe irradiance. However, the relative expression of *Nfkb1* was increased at 1064 nm, but not at 1270 nm. Serum anti-influenza IgG antibody titers increased early after immunization with 1064 nm, whereas with 1270 nm, there was not only an early response of antibody production but also persistence of antibody titers over the medium- to long-term. Thus, to our knowledge, we show for the first time that 1270 nm NIR light induces ROS and ATP production in mitochondria as photoreceptors, initiating a cascade of laser adjuvant effects for intradermal vaccines. Additionally, we demonstrate that there are wavelength-specific variations in the mechanisms and effects of laser adjuvants. In conclusion, CW1270 nm NIR light is expected to be clinically applicable as a novel laser adjuvant that is equivalent or superior to 1064 nm NIR light, because it can be operated at low energy and has a wavelength-specific adjuvant effect with medium- to long-lasting antibody titer.

## Introduction

The energy of light has various physiological effects when irradiated on living organisms; such effects are referred to as photobiomodulation (1). The mechanisms underlying this phenomenon have been proposed to include the photothermal effect induced by the conversion of photon energy into heat (2), the photoacoustic effect induced by the physical expansion and acoustics of tissues and cells (3), and the photochemical effect associated with the production of chemical substances (4, 5); however, the detailed mechanisms have not been elucidated. Because the absorption spectrum of photoreceptors in living organisms varies, the amount of photon energy absorbed by the organism varies with the wavelength of light and the density of photoreceptors in the tissue (6–8), suggesting that this can cause wavelength-specific photobiomodulation.

The development of laser technology began in the 1960s (9). A technique was developed to elicit an adjuvant effect—the laser adjuvant—mainly in the case of intradermal vaccination, by irradiating the skin with light under specific conditions using a laser as a light source (10, 11). Laser adjuvants can be classified as invasive or noninvasive. The former is considered to have an adjuvant effect by physical cauterization and transpiration of the skin, resulting in a healing response of the skin and local delivery of vaccine antigens through physically penetrated pores. Noninvasive laser adjuvants are thought to act on the previously mentioned photobiomodulation, and preclinical vaccine adjuvant studies using lasers of different wavelengths have partially, but not exclusively, validated different mechanisms (12).

Our group has been focusing on the wavelength range from 1000 nm to 1200 nm, which is not easily absorbed by melanosomes, regarding its application for all the human population (10, 11). In particular, we have been investigating the mechanism of laser adjuvant using a 1064 nm continuous wave (CW) 5 W/cm^2^ (13–15). Kimizuka et al. showed that bone marrow-derived mast cells generate reactive oxygen species (ROS) upon laser irradiation, which results in the release of chemokines, such as CCL2 and CCL20, and promotes migration of migratory dendritic cells (mDC) to the local lymph node, thereby, eliciting the adjuvant effect, suggesting that mast cells play a key role in laser adjuvant (15). However, the physiological activity of near-infrared red (NIR) light at the cellular level is still unclear, and it is not known how the light acts on mast cells to elicit the adjuvant effect.

Kimizuka et al. also demonstrated that NIR light at 1258 and 1301 nm has the similar adjuvant effect as NIR light at 1064 nm in an influenza vaccine model (16). Particularly, NIR light in the 1200 nm range can elicit equivalent adjuvant effects with a low laser irradiance of 40% of that of 1064 nm (1064 nm: 5 W/cm^2^, 1258 nm: 2 W/cm^2^). This means that more vaccinations can be performed with the same energy. In view of the future clinical applications, further research is required for the application of laser adjuvants using energy-efficient NIR light in the 1200 nm range.

Thus, in this study, we used 1270 nm NIR light as a novel laser adjuvant to investigate its safety, physiological activity on mast cells, biological activity on mouse skin, and durability of the vaccine adjuvant effect over the medium- to long-term in the influenza vaccine mouse model. We have, for the first time, identified mitochondria in mast cells as an important source of intradermal ROS production for NIR laser adjuvants and reveal the mechanism that leads to ATP production.

## Materials and methods

### Animals

A total of 161 female 8-week-old C57BL/6J mice (Japan SLC, Shizuoka, Japan) were used for all animal experiments. The animals were conditioned to the breeding room environment for at least 1 week before they were allowed to participate in the experiment. We used a ketamine/xylazine mixture to anesthetize the mice and carbon dioxide for euthanasia in our animal experiments. Animal experiments were conducted after obtaining approval from the Ethics Committee of the National Defense Medical College (Ethics Committee approval numbers: 18027, 18088).

### Laser illumination

**Figure 1:**
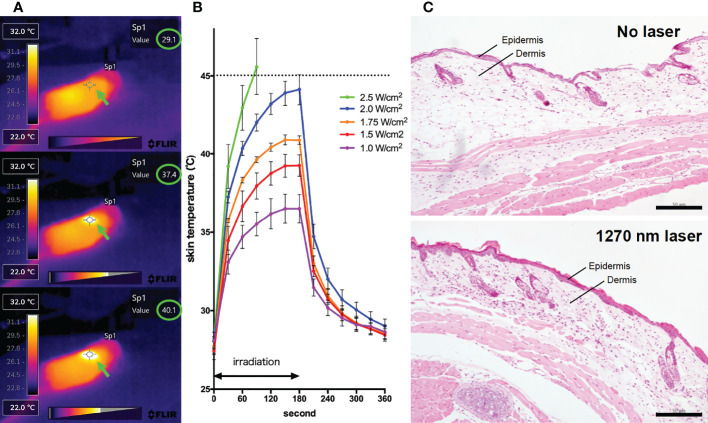
Effect of the near-infrared (NIR) 1270 nm laser adjuvant on skin tissue. **(A)** Representative images for measurement of the mice skin surface temperature using an infrared camera. Irradiated areas (arrows) and detected temperatures (circles) are shown. **(B)** Dose-temperature responses of the continuous wave 1270 nm laser for 180 s irradiation in mouse skin. Temperature represents the maximal skin surface temperature of irradiated center. Error bars show means ± s.e.m. **(C)** Microscopic assessment of skin damage and inflammatory infiltration after laser treatment. Representative images of hematoxylin-eosin-stained skin tissue are presented. In the laser group, epidermis, dermis, and deeper layers were intact. The scale bar represents 50 μm.

For the 1270 nm laser, a tunable fiber-coupled diode laser (Veralase, MA, USA), providing different wavelengths from 1260 to 1290 nm, was used. To emit 1270 nm CW laser, we set temperature at 20°C and both time on/pulse and time off/pulse at 0 millisecond. For the 1064 nm laser, an experimental semiconductor laser system (UNITAC, Hiroshima, Japan) was used, and the light source was kept constant at 37.5°C with a Peltier driver (JUST Co., Ltd., Tokyo, Japan) to stabilize the wavelength. In animal experiments, the diameter of the laser irradiation was 5 mm, and the laser was irradiated for 1 minute on mouse skin that had been shaved 2 days before irradiation. During irradiation, the temperature of the mouse skin surface was monitored using thermography (Teledyne FLIR, OR, USA) (**Figure 1A**). To determine the noninvasive irradiance of the 1270 nm laser, we previously assessed skin temperature during irradiation (**Figure 1B**) and pathological findings (**Figure 1C**) using 19 mice (13, 16). For preparation of skin pathology sections, the heart of mice in the no-laser and laser (1270 nm 2 W/cm^2^ for 3 min: the maximum irradiance at which the skin surface temperature during irradiation does not exceed 45°C) groups were perfused with 4% paraformaldehyde (PFA; FUJIFILM Wako, Osaka, Japan), 4 days after treatment. Thereafter, skin, including the irradiated area, was collected, and skin sections were stained with hematoxylin and eosin. Based on the preliminary results of these experiments, the noninvasive irradiance of the 1270 nm laser was determined to be 2 W/cm^2^. The noninvasive irradiance of the 1064 nm laser was set to 5 W/cm^2^, as reported previously (13).

### Detection of ROS in cultured mouse mast cells

Mouse mastocytoma cell line P-815 was provided by the RIKEN BRC through the National BioResource Project of the MEXT/AMED, Japan. The Roswell Park Memorial Institute (RPMI) 1640 medium (Thermo Fisher Scientific, MA, USA), supplemented with 10% fetal bovine serum (FBS) and antibiotic-antimycotic solution (Thermo Fisher Scientific, MA, USA) containing 100 µg/mL of streptomycin, 0.25 µg/mL of amphotericin B, and 100 units/mL of penicillin, was used for culture. Confluent P-815 cells were rinsed and resuspended in RPMI 1640 Medium, no phenol red (Thermo Fisher Scientific, MA, USA) and seeded at 1.8 × 10^5^ cells/well in 48-well plates (IWAKI, Shizuoka, Japan). We used RPMI 1640 medium without phenol red because phenol red absorbs light (17). 2′,7′-Dichlorodihydrofluorescein diacetate (H2DCFDA; Thermo Fisher Scientific, MA, USA) was added at a final concentration of 1 µM; the plate temperature was kept constant at 37°C using a hot plate (TOKAI HIT, Shizuoka, Japan) and the cells were irradiated with 1270 nm CW laser at 200 or 300 mW/cm^2^ for 1 minute. After irradiation, the cells were incubated in a CO_2_ incubator for 15 minutes, rinsed three times with Dulbecco’s phosphate-buffered saline (DPBS; Thermo Fisher Scientific, MA, USA) pre-warmed to 35°C, and analyzed with FACS Canto II (BD, NJ, USA) (50,000 events, FITC channel, excitation: 488 nm, detection: 535 nm). The FITC channel was used to detect the highly fluorescent 2′,7′-dichlorofluorescein (DCF; excitation: 492–495 nm/emission: 517–527 nm), produced by the oxidation of nonfluorescent H2DCFDA by ROS. As a positive control, hydrogen peroxide (H_2_O_2_) (FUJIFILM Wako, Osaka, Japan) was used at a final concentration of 500 µM. Mean fluorescence intensity (MFI) was calculated using the FlowJo v10 (BD, NJ, USA), and the MFI ratio of each group was calculated relative to the MFI of the no-laser group.

### Detection of ATP in cultured mouse mast cells and isolated mitochondria

For ATP detection, the luciferin-luciferase reaction (Lucifell HS Set; Kikkoman, Tokyo, Japan) was used. Confluent P-815 cells cultured in RPMI supplemented with 10% FBS in T75 flasks were rinsed twice with 1×PBS (Thermo Fisher Scientific, MA, USA) and resuspended in 200 µL of 1×PBS. Cell suspensions were seeded at 50 µL/well in 96-well plates (96 well assay plate, white plate, clear bottom with lid; Corning, NY, USA) with a constant temperature of 37°C on a hot plate. One sample at a time, 50 µL of ATP extraction reagent was mixed, and 20 seconds later, 100 µL of luminescent reagent was quickly added and fluorescence was measured using a microplate reader (SpectraMax iD5; Molecular devices, Tokyo, Japan). In the laser irradiation group, ATP extraction was performed immediately after irradiation with 1270 nm CW laser at 200 and 300 mW/cm^2^ for 1 minute.

For mitochondria isolation, the Mitochondria Isolation Kit for Cultured Cells (Thermo Fisher Scientific, MA, USA) was used. Mitochondria were isolated from 2.0 × 10^7^ P-815 cells using the Reagent-based method based on the manufacturer’s instructions. Before starting the experiment, we confirmed the presence of mitochondria in the obtained pellets using electron microscopy. Pellet was resuspended in the provided Reagent C 200 µL, and the fluorescence intensity was measured using the same method as for whole cells.

### Real-time RT-PCR of chemokine expression in mouse skin

A total of 23 mice were used in replicate experiments. The 1064 and 1270 nm CW laser were irradiated at 5 and 2 W/cm^2^, respectively, at four spots on the back of the dehaired mice, without overlap. Six hours after irradiation, 5 mm diameter skin including the irradiated area was stripped and soaked in RNAlater Stabilization Solution (Thermo Fisher Scientific, MA, USA). The collected skin was shredded into strips of tiny pieces using a pair of scissors, and homogenized in TRIzol Reagent (Thermo Fisher Scientific, MA, USA), using a Bullet Blender (NEXT ADVANCE, NY, USA). RNA extraction and its reverse transcription to cDNA were performed as described in our previous report (15). Primers used were *Ccl2*, *Ccl20*, *Nfkb1*, and *Nfkb2* (RT_2_ qPCR Primer Assays; QIAGEN, Venlo, Netherlands) with guaranteed amplification efficiency, and real-time RT-PCR was performed using a LightCycler 480 System (Roche, Basel, Switzerland). The relative expression levels of each chemokine were compared using the delta-delta Ct method with *Actb* (RT_2_ qPCR Primer Assays; QIAGEN, Venlo, Netherlands) as the reference gene (18). We had earlier confirmed the stable expression of *Actb* with or without laser irradiation.

### Protein extraction from mouse skin and cytokine ELISA

A total of 25 mice were used in replicate experiments. As mentioned above, the 1270 nm CW laser was irradiated at 2 W/cm^2^ at four spots on the back of dehaired mice, without overlap. The 5 mm diameter skin including the irradiated area was collected after euthanasia in the non-irradiated group (0H) and in the 6H, 9H, and 12H after-irradiation groups of mice. Collected skin was soaked in 1× PBS (Thermo Fisher Scientific, MA, USA) mixed with Halt Protease Inhibitor Cocktail (100X) (Thermo Fisher Scientific, MA, USA) on ice. The collected skin was shredded into strips of tiny pieces using a pair of scissors, and homogenated using a Bullet Blender (NEXT ADVANCE, NY, USA). Triton X-100 (Nacalai Tesque, Kyoto, Japan) was added to the homogenized solution, which was then frozen in liquid nitrogen. After thawing, the samples were centrifuged at 10,000 × *g* and the supernatant was filtered (Millex Syringe Filter, 0.22 µm; Merck Millipore, MA, USA). The amount of total protein extracted was determined using the Pierce Rapid Gold BCA Protein Assay Kit (Thermo Fisher Scientific, MA, USA). To quantify CCL2 and CCL20 proteins, the DuoSet ELISA kit (R&D Systems, MN, USA) was used. The CCL2 and CCL20 levels were corrected for the total protein extracted, and changes in intradermal chemokine levels over time after laser irradiation were analyzed.

### Immunofluorescence assay of mouse skin

Two mice per experiment were used, and the experiment was repeated twice for a total of four mice. Mouse ears were shaved and depilated 2 days before the tissue preparation. Depilated ears were treated with the NIR laser as described above. Six hours after the treatment, the hearts of anesthetized mice were perfused with 4% PFA (FUJIFILM Wako, Osaka, Japan). Mice ears were harvested and fixed in 4% PFA for an additional 4 hours. Ear samples were permeabilized and blocked with 0.3% Triton X-100 (Nacalai Tesque, Kyoto, Japan) and 0.5% Donkey serum albumin (Jackson Immuno Research, PA, USA) in 1× PBS (Thermo Fisher Scientific, MA, USA) overnight at 4°C. Samples were then incubated with a primary antibody to lymphatic vessel endothelial hyaluronan receptor 1 (Lyve-1; 1:400, rabbit polyclonal; Relia Tech, MN, USA) overnight at 4°C, followed by incubation with donkey anti-rabbit IgG (1:200, Jackson Immuno Research, PA, USA) at room temperature for 3 hours. Prolong Gold Antifade Mountant (Thermo Fisher Scientific, MA, USA) was added to coverslipped sections. We randomly sampled three to four photographic imaging stacks (900 × 900 μm^2^, 20–30 μm thick) for each ear using a confocal microscope (SP8; Leica Microsystems, Wetzlar, Germany) and visualized morphological changes in Lyve-1-positive lymphatic vessels using the Leica Application Suite Software (Leica Microsystems, Wetzlar, Germany) (**Figure 5A**).

### Mouse influenza vaccine models

A total of 90 mice were used in the replicate experiments (**Table 1**). First, 2 days prior to immunization, the backs of the mice were shaved and dehaired using a hair removal agent (Nair; Church & Dwight, NJ, USA) as we have previously reported (15). For the laser group, 10 µL of antigen solution was immediately injected intradermally (ID) after 1 minute of laser irradiation. A/PR/8/34 inactivated whole-particle influenza virus (1 µg in 10 µL saline, Charles River, MA, USA) was used as the antigen. For the Alum group, aluminum hydroxide (Imject Alum; Thermo Fisher Scientific, MA, USA) was mixed 1:1 with the vaccine. In the intramuscular (IM) injection group, injections were administered in the muscle of the right femur without laser irradiation. Blood samples were obtained at 7, 14, 21, 28, 56, 84, and 112 days after immunization.

**Table 1:** Characteristics of each group in mouse influenza vaccine models.

Group	Vaccine	Administration route	Adjuvant	Number
Saline	No vaccine	Intradermally	None	14
ID only	A/PR/8/34 inactivated whole-particle influenza virus			14
1064 nm laser			1064 nm NIR 5 W/cm^2^ 1 min	18
1270 nm laser			1270 nm NIR 2 W/cm^2^ 1 min	16
Alum			Aluminum hydroxide	14
IM		Intramuscular	None	14

### Anti-influenza antibody titers

Sera from all 90 immunized mice were included. Serum anti-influenza antibody titers (IgG, IgG1, and IgG2c) in each serum sample were analyzed using ELISA. Ninety-six-well plates (Thermo Fisher Scientific, MA, USA) were coated with 100 ng of the same antigen used for immunization. After applying the serially diluted samples, secondary antibodies IgG (Sigma, MO, USA), IgG1 (Southern Biotech, AL, USA), and IgG2c (Southern Biotech, AL, USA) were reacted with the samples. One-Step Ultra TMB-ELISA Substrate Solution (Thermo Fisher Scientific, MA, USA) was added, and the reaction was stopped with 10% sulfuric acid (FUJIFILM Wako, Osaka, Japan). Immediately thereafter, the optical density (OD) values at 650 and 450 nm were measured with a microplate reader. The subtracted OD values were plotted, and the dilution concentration at the inflection point was designated as the titer value.

### Influenza virus challenge study

All 90 mice immunized, as mentioned above, were used. On day 112 after immunization, mice were challenged intranasally with 30 µL of live influenza virus A/PR/8/34 (Charles River, MA, USA) at a dose that would be 5 × 10^5^ 50% egg infectious dose mixed with saline. The survival and body weight were monitored until day 15 post-challenge. As in our previous study (15), we considered the experimental endpoint for ethical reasons if there was hunched posture, ruffled fur, or >10% weight loss, or if the animal was unable to take water or food.

### Statistical analysis

The normal distribution was checked with the Shapiro–Wilk test. Because the data were not found to be normally distributed, we performed non-parametric Mann–Whitney and Kruskal–Wallis tests. The Dunn’s multiple comparison test was performed as a *post hoc* test. GraphPad Prism version 9 (GraphPad Software, CA, USA) was used for analysis with a significance level of 0.05.

## Results

### CW 1270 nm NIR light promotes ROS production in cultured P-815 cells

**Figure 2:**
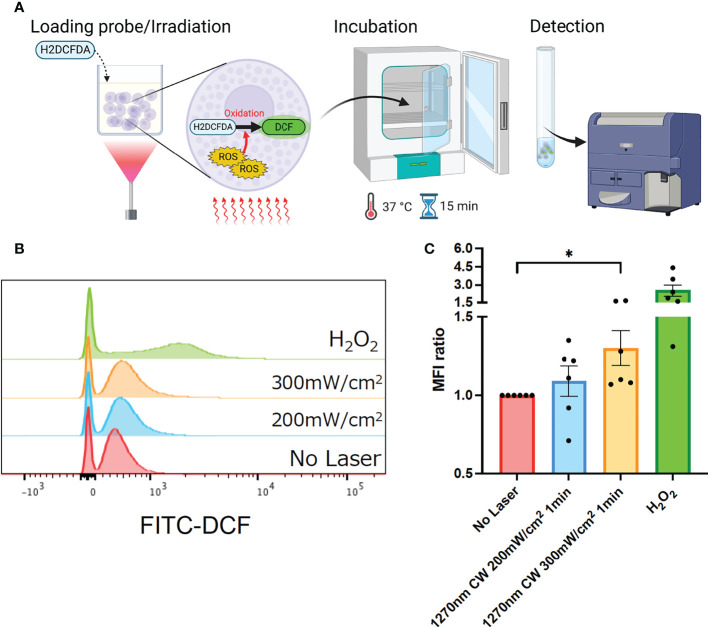
Generation of reactive oxygen species (ROS) induced by the near-infrared (NIR) laser treatment of cultured mast cells. **(A)** Release of ROS by the NIR laser treatment was assesssed by ROS-sensitive fluorescence probe, H_2_DCFDA, in P-815 *in vitro*. **(B)** P-815 cells were treated with the NIR laser at a power of 200 or 300 mW/cm^2^ for 1 min. ROS-reacted DCF-dependent fluorescence was measured using flow cytometry. **(C)** Fold increase in DCF+ population compared with that in no-laser control was calculated for each condition. Kruskal–Wallis test with Dunn’s multiple comparisons test (No Laser vs. 200 mW/cm^2^, 300 mW/cm^2^, *p<0.05. *n* = 6 per group), Error bars show mean ± s.e.m.

Irradiation of skin with NIR light has been reported to induce ROS production in skin tissue (15). In addition, mast cells in the skin are known to be a regulator of the immune system (19), and ROS production is known to have an important role in the mechanism of laser adjuvant effects of intradermal vaccines (15). Therefore, we examined whether the irradiation of a novel wavelength, 1270 nm NIR light, induces cellular ROS production in cultured P-815 cells. P-815 cells were irradiated at 200 and 300 mW/cm^2^ for 1 minute, and ROS-reacted DCF-dependent fluorescence was measured using flow cytometry (**Figure 2A**). H_2_O_2_-treated cells were used as a positive control. As we reported previously (15), this system could detect ROS constantly generated through aerobic metabolism, and DCF fluorescence-positive population was identified in the laser-treated group. The CW 1270 nm 300 mW/cm^2^ laser treatment significantly increased the ROS-related DCF+ population in cultured mast cells (**Figures 2B, C**, *p* = 0.019) compared with that in the non-laser-treated control group. MFI increased in an irradiance-dependent manner compared with that in the non-laser-treated control group (**Figure 2C**). These data demonstrate that the CW 1270 nm NIR light also induces ROS generation in mast cells.

### CW 1270 nm NIR light enhances ATP production in cultured P-815 cells and isolated mitochondria

**Figure 3:**
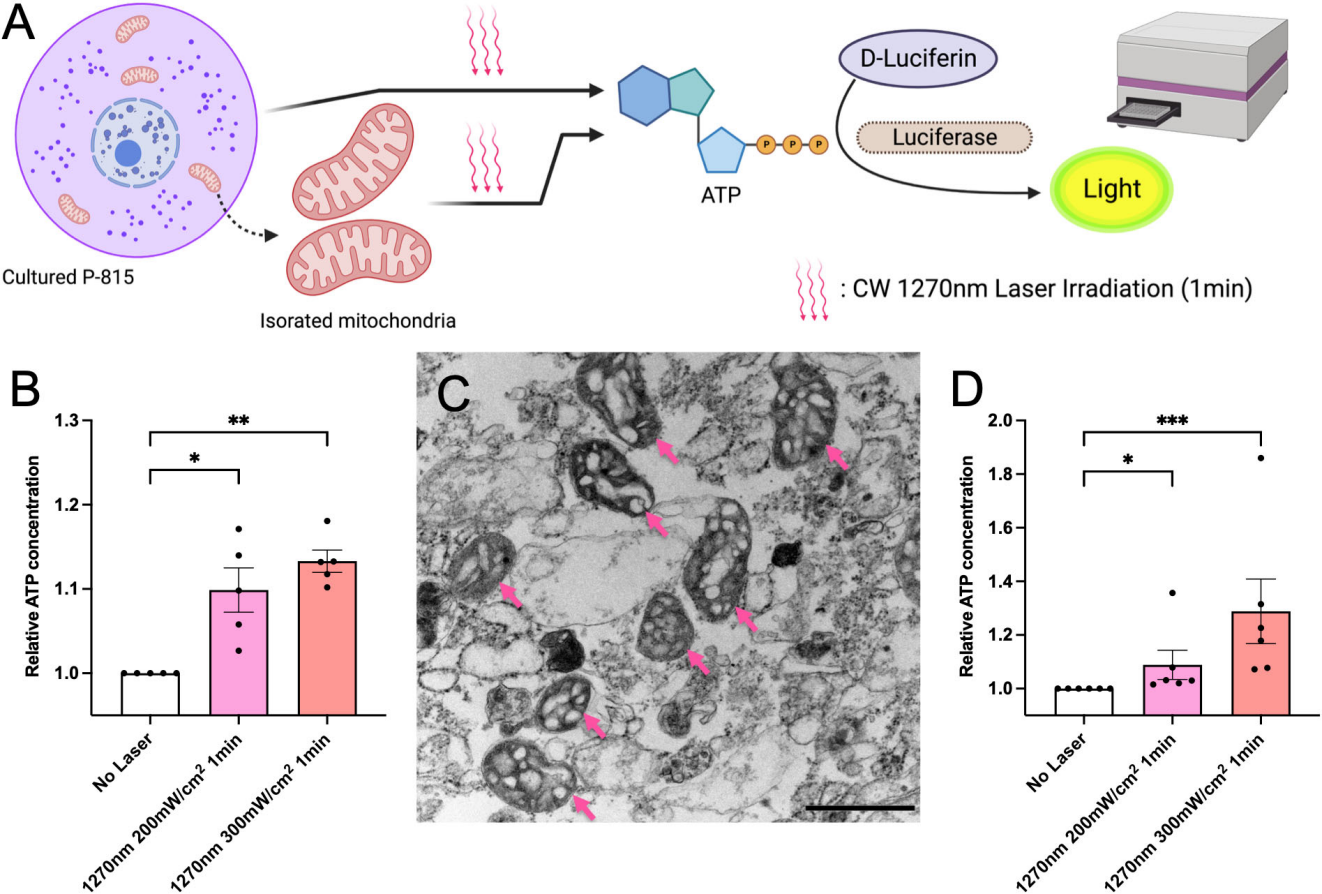
ATP generation induced by the near-infrared (NIR) laser treatment of cultured mast cells. **(A)** Release of ATP by the NIR laser treatment was assesssed using the luciferase luciferin reaction. **(B)** Fold increases in luminescence intensity using the luciferase reaction in cultured P-815 *in vitro*. **(C)** Isolated mitochondria were confirmed using electron microscopy (arrow); the scale bar represents 0.1 μm. **(D)** Fold increases in luminescence intensity using the luciferase reaction in mitochondria isolated from cultured P-815. Kruskal–Wallis test with Dunn’s multiple comparisons test (No Laser vs. 200 mW/cm^2^, 300 mW/cm^2^, *p<0.05, **p<0.01, ***p<0.001, B: *n* = 5 per group, D: *n* = 6 per group), Error bars show mean ± s.e.m.

In eukaryotes, ROS is mainly produced in mitochondria as a byproduct of aerobic respiration, in which oxygen is used to produce energy (ATP) necessary for growth. Therefore, ATP is closely related to ROS production in the cell (20). In this study, we tested whether irradiation with CW 1270 nm NIR laser induced ATP production in the cells. P-815 cells were irradiated at 200 and 300 mW/cm^2^ for 1 minute, and ATP production was measured using the luciferin-luciferase reaction and shown as relative values for the non-laser-treated control group (**Figure 3A**). In P-815 whole cells, irradiation with 1270 nm NIR light enhanced ATP production, which was irradiance-dependent (**Figure 3B**). We then focused on the mitochondria, where ATP is produced (21). We isolated mitochondria from P-815 cells and irradiated them as well as whole cells with 1270 nm NIR light (**Figure 3C**), and ATP production was determined as relative values to the non-laser-treated control group. The results showed that ATP was significantly produced both at 1270 nm 200 mW/cm^2^ and 300 mW/cm^2^ as in whole cells (**Figure 3D**; *p =* 0.036 and *p <* 0.001, respectively). These results demonstrate that the effect of laser adjuvant *via* mast cells upon irradiation with CW 1270 nm NIR light is triggered by the production of ATP and ROS in the intracellular mitochondria, acting as photoreceptors.

### CW 1270 nm NIR light upregulates *Ccl2* and *Ccl20* chemokine mRNAs in mouse skin

**Figure 4:**
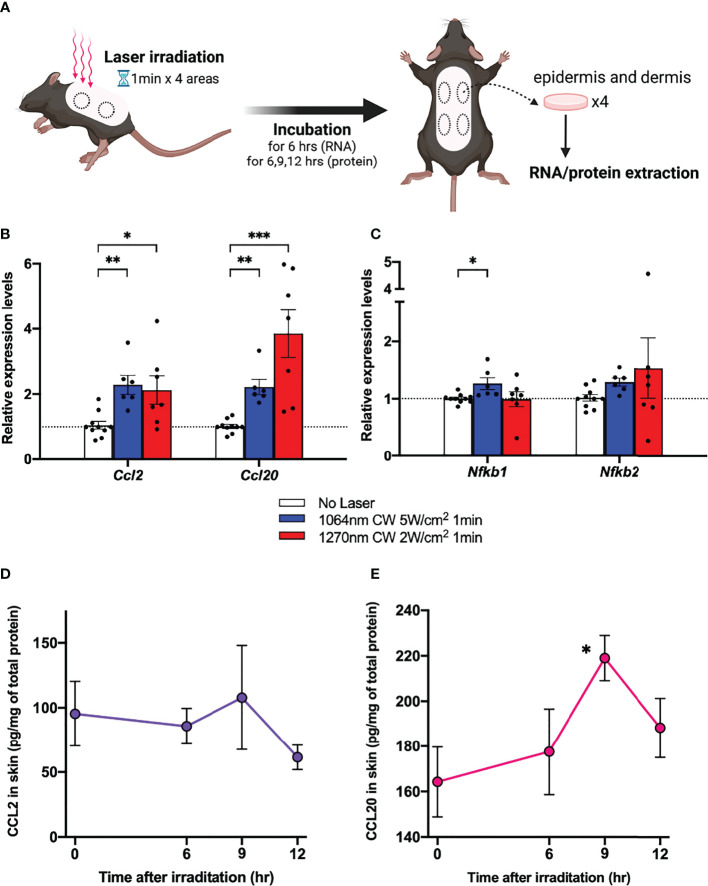
Expression of chemokines in skin in response to near-infrared (NIR) 1270 nm laser. **(A)** The effect of the CW NIR laser on the chemokine expression in the mouse back skin was measured 6 h following the CW NIR laser treatment using PCR (**B**: CCL2 and CCL20, **C**: Nfkb1 and Nfkb2). Kruskal–Wallis test with Dunn’s multiple comparisons test (No Laser vs. CW 1064 nm 5 W/cm^2^, CW 1270 nm 2 W/cm^2^, *p<0.05, **p<0.01, ***p<0.001, *n* = 10, 6, and 7 for No Laser, CW 1064 nm 5 W/cm^2^, and CW 1270 nm 2 W/cm^2^). Time course of chemokine protein levels at 6, 9, and 12 h after 1270 nm NIR laser irradiation (**D**: CCL2, **E**: CCL20). Mann–Whitney test (0 h vs. 6 h, 9 h, and 12 h for each, *p<0.05, *n* = 6, 6, 6, and 7 for 0, 6, 9, and 12 h). Error bar show mean ± s.e.m.

In a previous study, we found that the expression of *Ccl2* and *Ccl20* chemokine mRNAs was upregulated in mouse skin 6 h after 1064 nm laser irradiation (15). To investigate whether a similar mechanism is occurring at 1270 nm, we examined the *Ccl2* and *Ccl20* mRNA expression levels (**Figure 4A**). The relative expression level of *Ccl2* and *Ccl20* chemokine mRNAs was increased about 2- and 4-fold, respectively, in mouse skin 6 h after 1270 nm laser irradiation (**Figure 4B**). Next, we tested the relative expression levels of *Nfkb1* and *Nfkb2*, which encode p50 and p52, members of the NF-κB family of transcription factors that function at the core of the inflammatory response (22, 23), in relation to the enhancement in ATP production in mitochondria induced by laser irradiation. Although *Nfkb1* showed a significant increase in expression level in the 1064 nm laser group (*p* = 0.011), the expression level was not changed by the 1270 nm laser. On the contrary, *Nfkb2* was not significantly upregulated by the 1064 and 1270 nm lasers but tended to be upregulated by the 1270 nm laser (**Figure 4C**). Quantitative time course evaluation of proteins in the skin was performed for CCL2 and CCL20, the gene expression levels of which were increased after irradiation with the 1270 nm NIR laser. The production of both CCL2 (**Figure 4D**) and CCL20 (**Figure 4E**) peaked at 9 h post irradiation and was significantly higher for CCL20 than at baseline (0 h).

### 1270 nm NIR light dilates Lyve-1-positive lymphatic vessels in mouse skin

**Figure 5:**
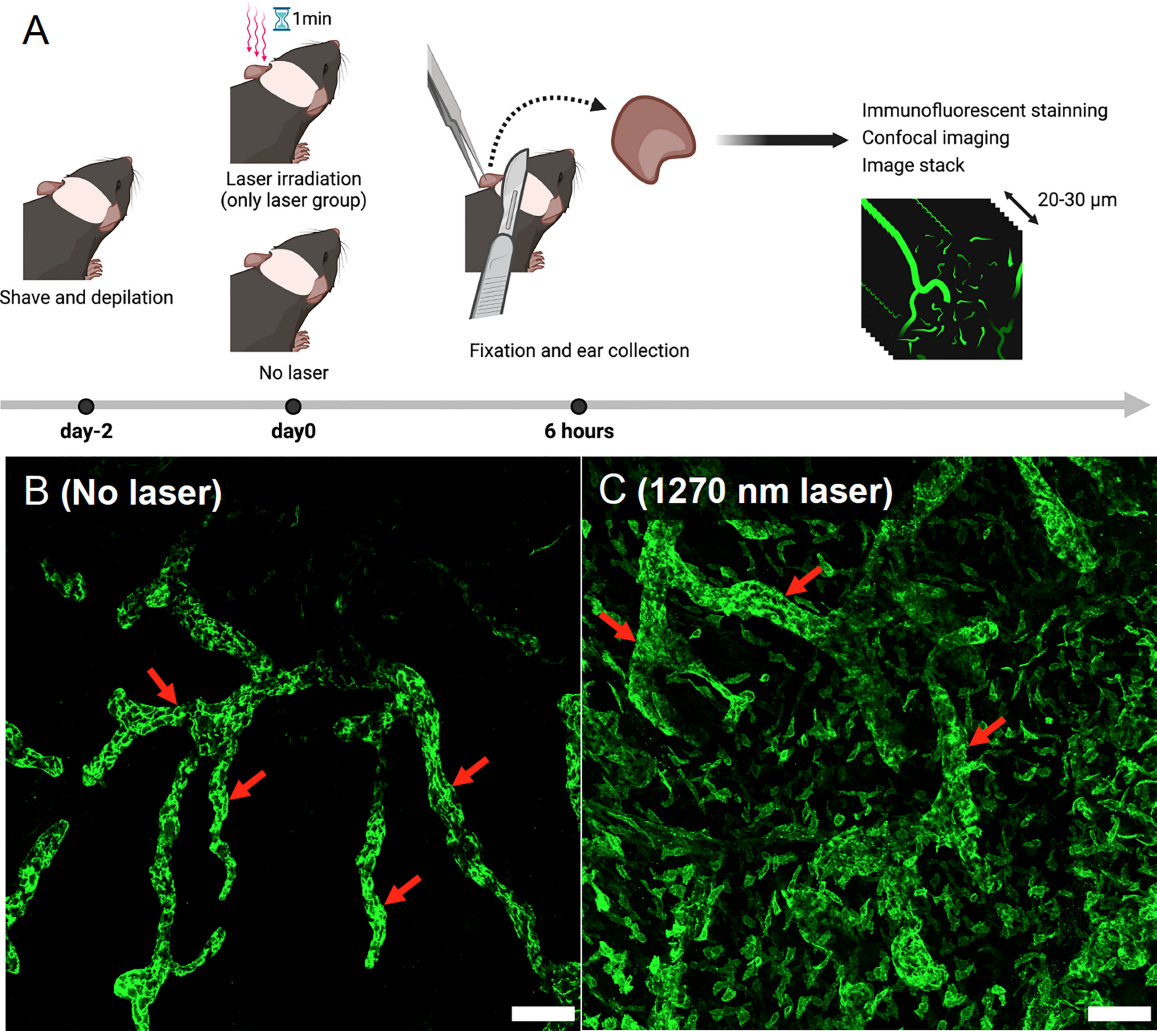
Evaluation of morphological changes in intradermal lymphatic vessels using immunofluorescence assay. **(A)** Immunofluorescence staining was performed on mouse ears 6 h after 1270 nm NIR laser irradiation, and images were taken with a confocal microscope. Morphological changes were visualized in stacks of the three-dimensional confocal images. **(B)** No-laser group. The main trunks of intradermal Lyve-1-positive lymphatic vessels (arrows) are imaged in green. **(C)** 1270 nm NIR laser group. The main trunk of intradermal Lyve-1 positive lymphatic vessels (arrows) is dilated, and microvessels are also dilated throughout the field of view. The scale bar represents 100 μm.

It is known that UV-B or 1064 nm NIR light induces morphological changes in intradermal lymphatic vessels and increases the density of the lymphatic network by dilating the capillary lymph vessels, which contributes to the migration of dendritic cells to the lymph nodes belonging to the skin (15, 24). Similarly, in the present study, both lymphatic main stem and capillary lymph vessels were dilated in the skin of mice 6 hours after irradiation with 1270 nm NIR light (**Figures 5B, C**).

### Vaccine adjuvant effect with 1270 nm NIR laser produces medium- and long-term durable antibody titers

**Figure 6:**
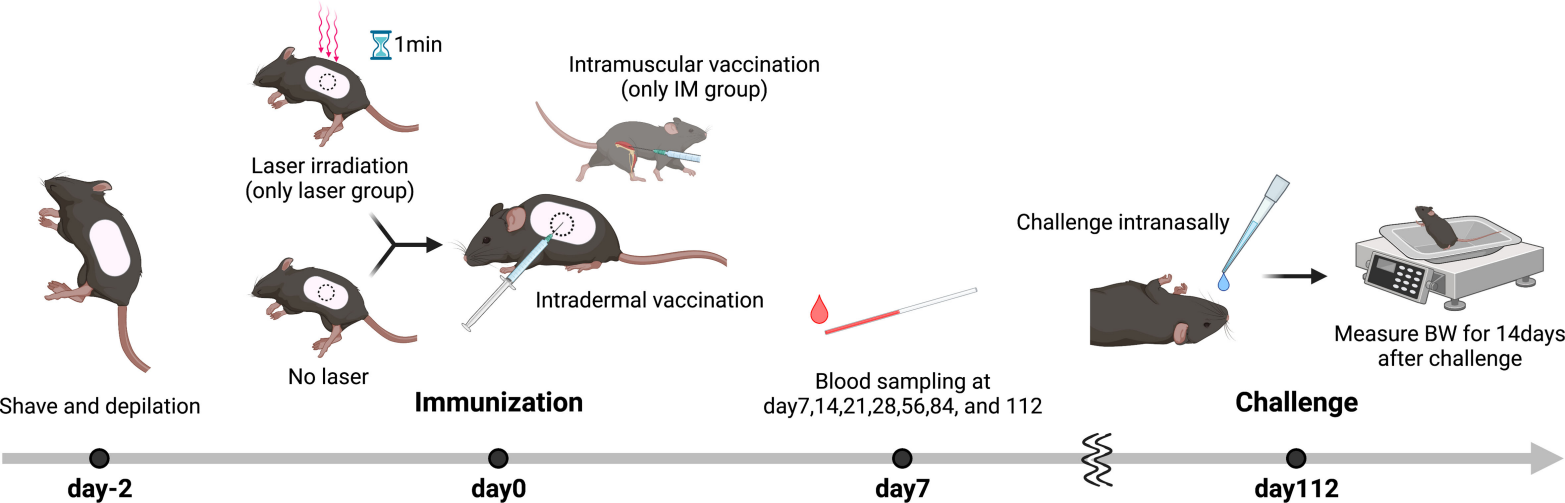
Influenza vaccination models with near-infrared (NIR) laser adjuvant. The back skin of wild type (C57BL/6J) mice were depilated with a commercial depilatory cream, 2 days before the NIR laser treatment and vaccination. Mice were vaccinated with 1 μg of whole inactivated influenza virus (A/PR/8/34) with or without the NIR laser exposure (1064 nm CW 5 W/cm^2^ for 1 min or 1270 nm CW 2 W/cm^2^ for 1 min) or alum intradermally (ID) or intramuscularly (IM). Longitudinal blood serum was collected *via* the tail vein at day 7, 14, 21, 28, 56, 84, and 112. Mice were challenged intranasally with homologous live influenza A/PR/8/34 virus at a dose of 5 × 10^5^ 50% egg infectious doses at day 112.

**Figure 7:**
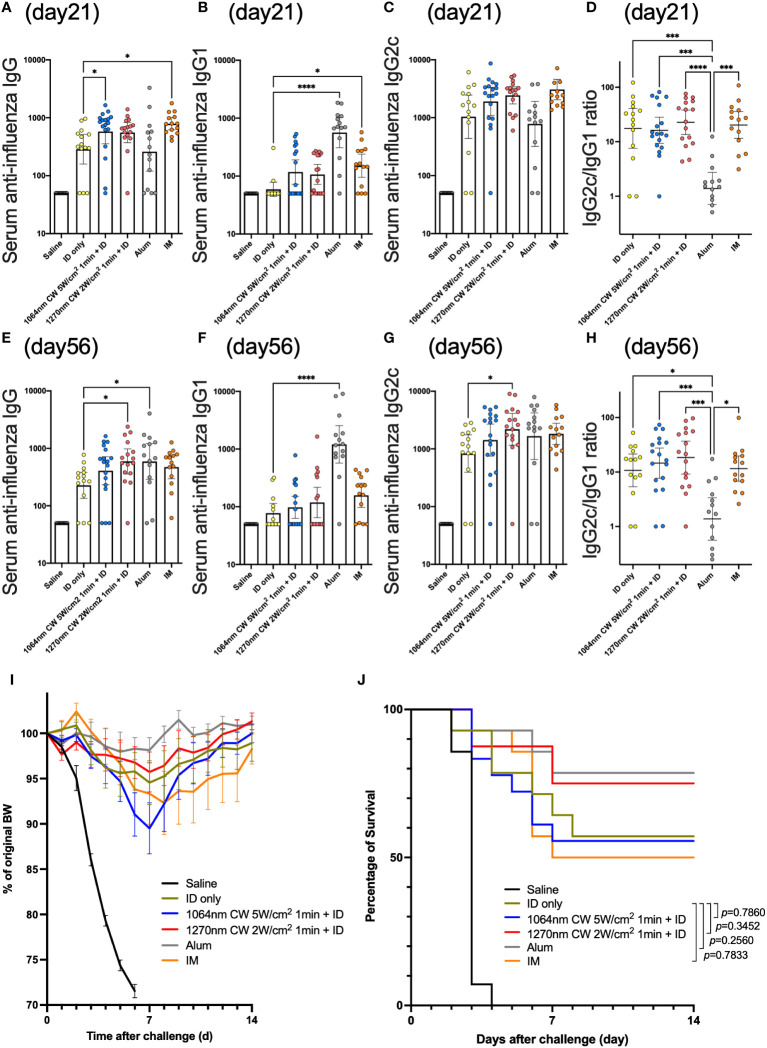
Effect of the near-infrared (NIR) laser adjuvant on anti-influenza immune responses. Serum anti-influenza specific **(A)** IgG, **(B)** IgG1, **(C)** IgG2c, **(D)** IgG2c/IgG1 ratio at day 21, and **(E)** IgG, **(F)** IgG1, **(G)** IgG2c, **(H)** IgG2c/IgG1 ratio at day 56. **(A–H)** *n* = 14, 14, 18, 16, 14, and 14 for no vaccine (saline), vaccine ID only (ID only), 1064 nm CW laser + vaccine ID, 1270 nm CW laser + vaccine, vaccine/alum ID (Alum), and vaccine IM, respectively. Results were pooled from three independent experiments and analyzed using the Kruskal–Wallis test followed by the Dunn’s multiple comparison test. (*p<0.05, ***p<0.001, ****p<0.0001 compared with ID only) Geometric mean with 95% CI. **(I)** The effect of the NIR laser adjuvant on body weight of vaccinated mice following viral challenge. Body weights were monitored daily for 2 weeks. Mean body weight ± s.e.m. *n* = 14, 14, 18, 16, 14, and 14 for no vaccine (saline), vaccine ID only (ID only), 1064 nm CW laser + vaccine ID, 1270 nm CW laser + vaccine, vaccine/alum ID (Alum), and vaccine IM, respectively. **(J)** Kaplan–Meier survival plots for 2 weeks following lethal influenza challenge; Gehan–Breslow–Wilcoxon test. **(I, J)** Results were pooled from three independent experiments.

In the influenza vaccine mouse model (**Figure 6**), serum anti-influenza IgG antibody titer was significantly increased in the IM and 1064 nm laser groups on day 21 (**Figure 7A**; *p* = 0.011 and *p* = 0.019, respectively). At this time, IgG1 was significantly increased in the Alum and IM groups (**Figure 7B**; *p <* 0.0001 and *p* = 0.030, respectively), and there was no significant difference in IgG2c levels between the groups (**Figure 7C**). IgG2c/IgG1 ratio, which reflects Th1-Th2 balance, was remarkably lower in the Alum group than in the other groups (**Figure 7D**). However, on day 56, IgG antibody titers tended to decrease in the IM and 1064 nm laser groups, whereas significantly higher titers were observed in the 1270 nm laser and Alum groups (**Figure 7E**; *p =* 0.025 and *p* = 0.018, respectively). At this time, IgG1 antibody titer was significantly higher only in the Alum group (**Figure 7F**; *p <* 0.0001) and IgG2c antibody titer was significantly higher only in the 1270 nm laser group (**Figure 7G**; *p* = 0.048). The IgG2c/IgG1 ratio on day 56 was lower in the Alum group than in the other groups, as in day 21 (**Figure 7H**).

In the challenge test, body weight decreased in all groups with a peak on day 7 post-challenge and tended to recover thereafter (**Figure 7I**). Survival rates tended to be higher in the Alum and 1270 nm laser groups but lower in the IM group; however, no group had significantly different mortality rates than the ID only group (**Figure 7J**).

## Discussion

In this study, for the first time to our knowledge, we focused on laser adjuvants using 1270 nm, the most energy-efficient CW NIR light, and found that NIR laser induced ROS and ATP production in mitochondria as photoreceptors (i.e., absorbers), which triggered a cascade of adjuvant effects, such as activation of dendritic cells (DCs) by increasing mRNA and protein expression of CCL2 and CCL20 and improvement of the traffic to affiliated lymph nodes by expansion of intradermal capillary lymph vessels, in the case of intradermal vaccines. In addition, using the same whole-particle intradermal influenza vaccine model, the laser adjuvant was characterized by wavelength-specific adjuvant effects, with a known early antibody induction effect at 1064 nm, whereas a medium- to long-term antibody durability was seen at 1270 nm. These mechanisms and characteristics are expected to provide important insights into the future clinical application of this adjuvant technology.

In principle, for light to have a biological effect, photons must be absorbed by photoreceptors in the living body (25). In this study, 1270 nm NIR light irradiation of P-815, a mouse mastocytoma, promoted the production of ROS and ATP, and mitochondria isolated from P-815 also generated ATP upon NIR light irradiation. This suggests that mitochondria function as photoreceptors of 1270 nm NIR light and may be the initiator of the cascade of vaccine adjuvant effects. It is generally believed that cytochrome c oxidase (CCO) in mitochondria acts as a photoreceptor in near-infrared light, which is much shorter than the wavelength we used, and its peak is in the wavelength range of 600 to 850 nm (26–28). However, in the wavelength range above 1000 nm, such as the 1270 nm NIR light we used, the details are unclear. Theoretically, NIR light at longer wavelengths (>1000 nm) has a lower absorbance on CCOs than the aforementioned wavelengths from 600 to 850 nm, but it is considered to be able to have an effect on CCOs and ion channels in deeper structures because light penetrates deeper structures due to less scattering (29–32). Additionally, because mast cells, which are essential for the NIR laser adjuvant effect above 1000 nm (15), used in this study, are generally located in the subepithelial region of the connective tissue surrounding blood cells, smooth muscle, mucosa, and hair follicles rather than on the skin surface (33), the use of NIR light above 1000 nm, which can reach deeper structures, is convenient for eliciting the adjuvant effect. However, it has also been shown that irradiation of human umbilical vein endothelial cells with NIR light at 1064 and 1270 nm generates nitric oxide (NO), a kind of ROS (34). NO inhibits the transport of electrons in the electron transport chain and increases oxygen consumption by elevating mitochondrial membrane potential, and the proton gradient is thought to ultimately lead to enhanced ATP production (28). In addition, NO also dilates lymphatic vessels (24, 35) and may contribute to the dilation of intradermal capillary lymph vessels as seen in this study. Thus, it is inferred that 1270 nm NIR light activates multiple cells, such as mast cells, keratinocytes (15), and endothelial cells (34), which are abundant in deep skin locations, and that the vaccine adjuvant effect is elicited by the interaction of these cells.

In the present study, the expression of the *Ccl2* and *Ccl20* chemokine mRNAs was upregulated in the skin of mice 6 h after irradiation with 1270 nm NIR light. This phenomenon was similar to our previous observation using a 1064 nm NIR light (15). In addition, this experiment was the first to quantitatively evaluate chemokines in the skin after 1270 nm NIR laser irradiation, showing increased concentrations of CCL20 and CCL2 in the skin 9 h after irradiation. Furthermore, 1064 nm NIR light enhances the production of CCL2 and CCL20 chemokines that activate Langerin+ and CD11b-/Langerin- mDCs and augment their migration to the skin-affiliated lymph nodes, and by convening CD11b+/Ly6C+ monocytes, which is thought to elicit the adjuvant effect (14). Although it is likely that the same phenomenon occurs with 1270 nm NIR light, it is also possible that there is wavelength-specific biological activity, and it will be necessary to confirm the functional role of DCs upon irradiation with 1270 nm NIR light in the future. For mRNAs associated with the NF-κB pathway, interestingly, *Nfkb1* was significantly increased at 1064 nm, but not at 1270 nm. On the contrary, *Nfkb2* showed no significant elevation in relative expression in both 1064 and 1270 nm, but there was a trend toward a slight increase in expression in 1270 nm. p50 and p52 encoded by *Nfkb1* and *Nfkb2*, respectively, are regulated by canonical and non-canonical NF-κB pathways (22, 23). Thus, it indicates that signaling through the canonical NF-κB pathway occurs at 1064 nm, but not at 1270 nm. Rather, the non-canonical NF-κB pathway may be activated more than the canonical NF-κB pathway in 1270 nm NIR light, suggesting that wavelength-specific intracellular signaling may be different from that in 1064 nm NIR light. NF-κB pathway is also associated with the behavior of DCs in innate immunity; in general, DCs sense infection and tissue damage and mature as antigen-presenting cells, which involves the canonical NF-κB pathway (22). However, DCs also express tumor necrosis factor receptor superfamily members, such as CD40 (36), lymphotoxin-beta receptor (37), and receptor activator of NF-κB (38, 39). This is thought to enable the stimulation of DCs *via* the non-canonical NF-κB pathway (40). These findings suggest that wavelength-specific differences in active signals in the NF-κB pathway may be one of the factors causing subtle differences in wavelength-specific biological activities of NIR light, including those in the behavior of DCs in innate immunity.

Examination of antibody titers using the influenza vaccine mouse model showed that IgG antibody titers in the 1270 nm laser group tended to be higher on day 21 than in the ID only group and were significantly higher on day 56. However, similar to our previous report (41), the 1064 nm laser group in the present study elicited an early antibody production response at day 21. Interestingly, a similar trend was observed during the entire observation period from day 7 to day 112 (**Figure S1**). The 1270 nm laser was more effective at eliciting durable antibody levels than at inducing an early humoral response, whereas the opposite was observed with the 1064 nm laser. This difference in medium- to long-term durability of antibody titers at different wavelengths was unexpected, and we consider the following as one of the reasons. In general, it is known that the long-term persistence of serum antibody titers is mediated by the persistent secretion of antibodies by terminally differentiated long-lived plasma cells (42). On the contrary, NF-κB-inducing kinase, a key mediator of the non-canonical NF-κB pathway that was possibly activated in the 1270 nm laser group, is considered important for the survival of terminally differentiated plasma cells and class-switched B cells (43). This may have contributed to the long-lasting antibody titers observed with 1270 nm NIR light. It is possible that slight differences in the wavelength-specific biological activity of NIR light are related to the characteristics of antibody production, and further studies are required to elucidate the detailed mechanisms. As for the IgG2c/IgG1 ratio, it was was significantly lower in the Alum group than in the other groups, similar to previous reports (15, 41). This appears to be due to IgG1-dominant antibody production caused by alum, a potent inducer of Th2-type immune responses (44). In contrast, the values in the 1270 nm laser group were similar to those in the ID only group, the 1064 nm laser group, and the IM group, which may induce a Th1-Th2 balanced adjuvant effect and may be applicable as an adjuvant with less allergic reactions. Challenge test showed that the 1270 nm laser group and the Alum group tended to have lower weight loss and higher survival rates than the other groups, which is proportional to the level of anti-influenza antibody titers at the time of the challenge. It is thought that the survival rate did not significantly change because the ID only group was used as the control, which makes the comparison test less likely to show a difference with the adjuvant group, and the difference may become significant as the sample size is increased in the future.

This study showed not only a medium- to long-term durability of antibody titers in the 1270 nm laser group, but also a trend toward an early response in antibody production. Therefore, the adjuvant effect can be achieved at lower energy than that of 1064 nm NIR light, and furthermore, the antibody elicitation ability is considered to be the same or higher. It is useful for dose sparing of antigens, as well as for operation to mass vaccination sites, as it requires less energy and is available to a larger number of people. For these reasons, CW 1270 nm NIR light could be suitable in terms of clinical applications of laser adjuvant technology. Our previous studies, as well as this study, have been validated using inactivated whole-particle vaccines, which are highly immunogenic. However, advances in vaccine production technology are expected to lead to a shift to more selective antigens, such as protein and mRNA vaccine (45), and the combination of these vaccines and laser adjuvants should also be considered. Additionally, recent studies have suggested that mucosal immunization with intranasal influenza live attenuated vaccines may enhance the response of tissue resident B and T cells in the respiratory tract (46), and the application of this technology in these new administration routes is also expected.

In conclusion, we demonstrate that the new laser adjuvant, 1270 nm NIR light, is absorbed by mitochondria, where the production of ATP may play a role in eliciting the adjuvant effect in intradermal vaccines.

## Data availability statement

The original contributions presented in the study are included in the article/**Supplementary Material**. Further inquiries can be directed to the corresponding author.

## Ethics statement

The animal study was reviewed and approved by National Defense Medical College Animal Experiment Ethics Review Committee.

## Author contributions

YMa and YK conceived and designed the experiments. YMa, YK, TS, EKo, EKi, YS, TOg, ST, and RN contributed to conducting experiments. SY and SK contributed to obtaining some reagents. YMa, TS, EKo, EKi, and TOg performed the analysis of samples. YK, TOg, and TK provided advice on experiments. YK, TOn, TK, MI, YMi, SK, and AK directed the study. YMa and YK wrote the manuscript. All authors contributed to the review and editing of the manuscript. All authors contributed to the article and approved the submitted version.

## Funding

This study was supported by GSK Japan Research Grant 2017 (YK), The Uehara Memorial Foundation 2018 (YK), Japan Society for the Promotion of Science (JSPS): Grant-in-Aid for Early-Career Scientists (project number: 19K16703) (YK), JSPS: Invitational Fellowships for Research (Short-term) 2020 (YK), Akaeda Medical Research Foundation 2020 (YK), 47^th^ Ohyama Health Foundation (YK), Defense Medicine Basic Research Program C 2021 (YMa), Defense Medicine Basic Research Program B 2022 (YK), and Japan Agency for Medical and Development (AMED) (Project number: 22fk0108647j0001) (YK).

## Acknowledgments

We are grateful to Y. Komatsu and K. Kozu (Division of Infectious Diseases and Respiratory Medicine, Department of Internal Medicine, National Defense Medical College) for taking care of the general affairs of this research. We thank T. Hirasawa, Y. Mayumi, and M. Miyashita (Department of Medical Engineering, National Defense Medical College) for technical support of laser set-up and provision of experimental environment. We created the schema in **Figures 2A**, **3A**, **4A**, **5A**, and **6** with BioRender.com (Agreement number: OG24BPIHE5, PL24BPHY3U, TC24HDU6CA, NX24HDSTRM, and PQ24BEV85M, respectively).

## Conflict of interest

The authors declare that the research was conducted in the absence of any commercial or financial relationships that could be construed as a potential conflict of interest.

## Publisher’s note

All claims expressed in this article are solely those of the authors and do not necessarily represent those of their affiliated organizations, or those of the publisher, the editors and the reviewers. Any product that may be evaluated in this article, or claim that may be made by its manufacturer, is not guaranteed or endorsed by the publisher.

## Supplementary material

Supplementary Figure 1 Longitudinal effect of the near-infrared (NIR) laser adjuvant on anti-influenza immune responses Serum anti-influenza specific **(A–G)** IgG, **(H–N)** IgG1, **(O–U)** IgG2c at day 7-112. *n* = 14, 14, 18, 16, 14, and 14 for no vaccine (saline), vaccine ID only (ID only), 1064 nm CW laser + vaccine ID, 1270 nm CW laser + vaccine, vaccine/alum ID (Alum), and vaccine IM, respectively. Results were pooled from three independent experiments and analyzed using the Kruskal–Wallis test followed by the Dunn’s multiple comparisons test. (**p*<0.05, ***p*<0.01, ****p*<0.001, *****p*<0.0001 compared with ID only) Geometric mean with 95% CI. Data of day 21 and 56 from are shown for comparison.
