# Corrigendum: 1270 nm near-infrared light as a novel vaccine adjuvant acts on mitochondrial photoreception in intradermal vaccines

**DOI:** 10.3389/fimmu.2024.1446711

**Published:** 2024-06-27

**Authors:** Yohei Maki, Toshihiro Kushibiki, Tomoya Sano, Takunori Ogawa, Eri Komai, Shusaku Takahashi, Etsuko Kitagami, Yusuke Serizawa, Ryosuke Nagaoka, Shinya Yokomizo, Takeshi Ono, Miya Ishihara, Yasushi Miyahira, Satoshi Kashiwagi, Akihiko Kawana, Yoshifumi Kimizuka

**Affiliations:** ^1^ Division of Infectious Diseases and Respiratory Medicine, Department of Internal Medicine, National Defense Medical College, Tokorozawa, Japan; ^2^ Department of Medical Engineering, National Defense Medical College, Tokorozawa, Japan; ^3^ Gordon Center for Medical Imaging, Department of Radiology, Massachusetts General Hospital, Charlestown, MA, United States; ^4^ Department of Global Infectious Diseases and Tropical Medicine, National Defense Medical College, Tokorozawa, Japan

**Keywords:** adjuvant, laser, light, mitochondria, vaccine, near-infrared, ROS- reactive oxygen species, ATP- adenosine triphosphate

**Error in Figure/Table**

In the published article, there was an error in **Figure 7** as published. “Day 28” was labeled in **Figures 7A–D** instead of “Day 21”, which is inconsistent with the main text and legend. The corrected **Figure 7** and its caption appear below.

**Figure 7:**
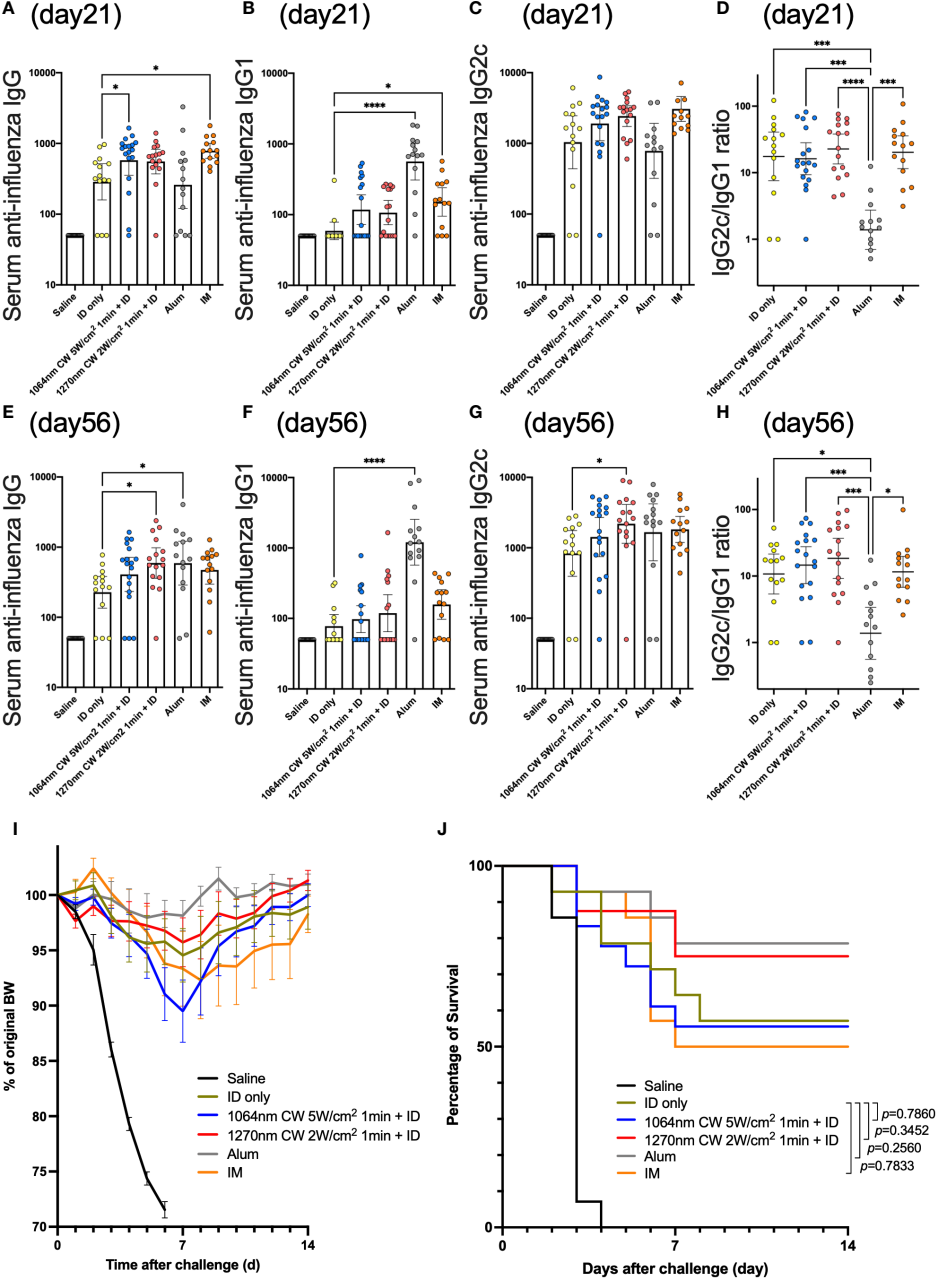
Effect of the near-infrared (NIR) laser adjuvant on anti-influenza immune responses. Serum anti-influenza specific **(A)** IgG, **(B)** IgG1, **(C)** IgG2c, **(D)** IgG2c/IgG1 ratio at day 21, and **(E)** IgG, **(F)** IgG1, **(G)** IgG2c, **(H)** IgG2c/IgG1 ratio at day 56. **(A–H)** *n* = 14, 14, 18, 16, 14, and 14 for no vaccine (saline), vaccine ID only (ID only), 1064 nm CW laser + vaccine ID, 1270 nm CW laser + vaccine, vaccine/alum ID (Alum), and vaccine IM, respectively. Results were pooled from three independent experiments and analyzed using the Kruskal–Wallis test followed by the Dunn’s multiple comparison test. (*p<0.05, ***p<0.001, ****p<0.0001 compared with ID only) Geometric mean with 95% CI. **(I)** The effect of the NIR laser adjuvant on body weight of vaccinated mice following viral challenge. Body weights were monitored daily for 2 weeks. Mean body weight ± s.e.m. *n* = 14, 14, 18, 16, 14, and 14 for no vaccine (saline), vaccine ID only (ID only), 1064 nm CW laser + vaccine ID, 1270 nm CW laser + vaccine, vaccine/alum ID (Alum), and vaccine IM, respectively. **(J)** Kaplan–Meier survival plots for 2 weeks following lethal influenza challenge; Gehan–Breslow–Wilcoxon test. **(I, J)** Results were pooled from three independent experiments.

The authors apologize for this error and state that this does not change the scientific conclusions of the article in any way. The original article has been updated.

