# Supplementary figures and images for: 1270 nm near-infrared light as a novel vaccine adjuvant acts on mitochondrial photoreception in intradermal vaccines
Source: Front Immunol. 2022 Nov 10;13:1028733. doi: 10.3389/fimmu.2022.1028733 (PMC9684730; doi:10.3389/fimmu.2022.1028733)

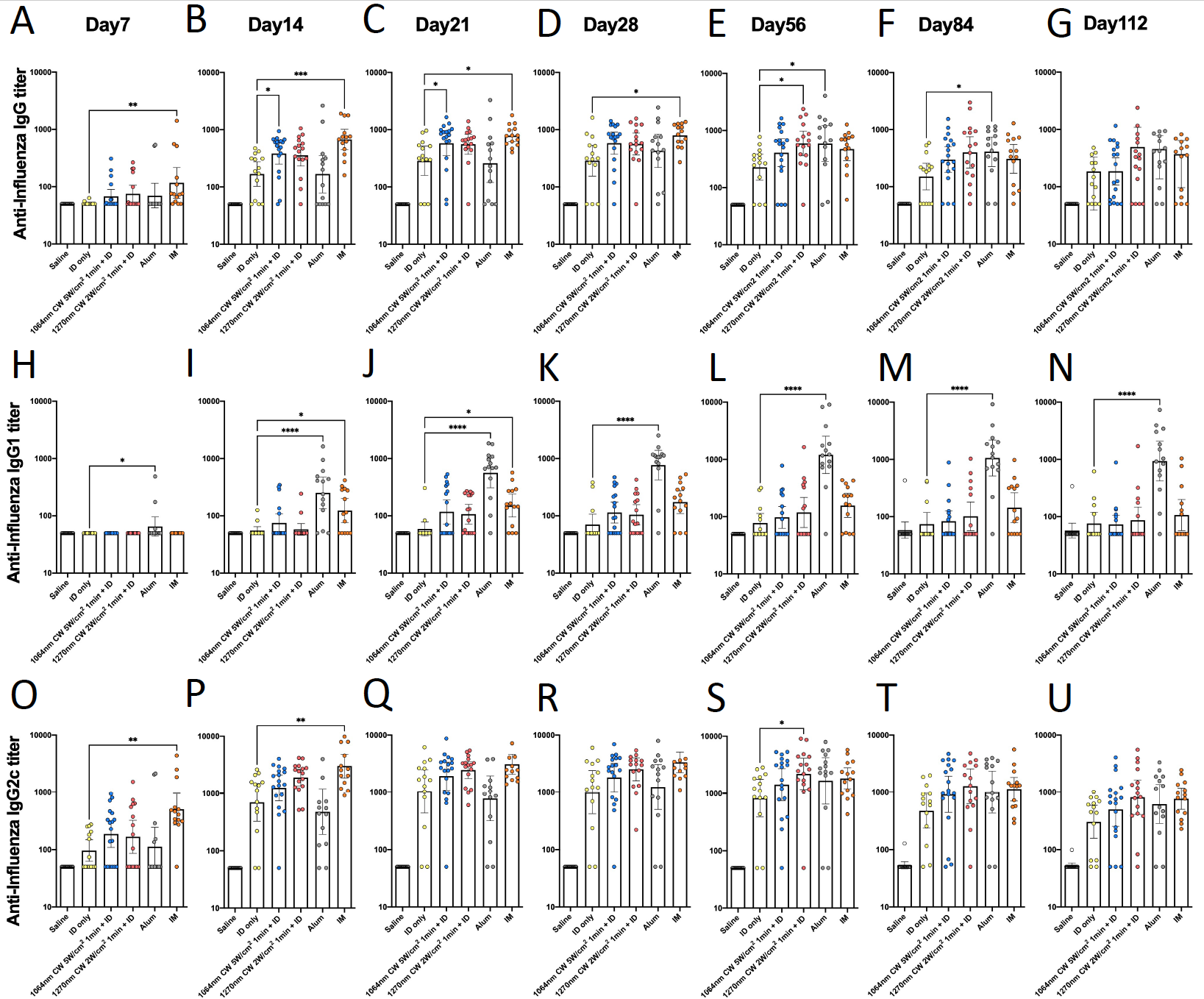

Supplement: Supplementary Figure 1 — Longitudinal effect of the near-infrared (NIR) laser adjuvant on anti-influenza immune responses Serum anti-influenza specific (A–G) IgG, (H–N) IgG1, (O–U) IgG2c at day 7-112. n = 14, 14, 18, 16, 14, and 14 for no vaccine (saline), vaccine ID only (ID only), 1064 nm CW laser + vaccine ID, 1270 nm CW laser + vaccine, vaccine/alum ID (Alum), and vaccine IM, respectively. Results were pooled from three independent experiments and analyzed using the Kruskal–Wallis test followed by the Dunn’s multiple comparisons test. (*p<0.05, **p<0.01, ***p<0.001, ****p<0.0001 compared with ID only) Geometric mean with 95% CI. Data of day 21 and 56 from are shown for comparison. [file Image_1.jpeg]
